# Prediction signals in the cerebellum: Beyond supervised motor learning

**DOI:** 10.7554/eLife.54073

**Published:** 2020-03-30

**Authors:** Court Hull

**Affiliations:** Department of Neurobiology, Duke University School of MedicineDurhamUnited States; University of California, BerkeleyUnited States; University of California, BerkeleyUnited States

**Keywords:** cerebellum, motor learning, neural circuits

## Abstract

While classical views of cerebellar learning have suggested that this structure predominantly operates according to an error-based supervised learning rule to refine movements, emerging evidence suggests that the cerebellum may also harness a wider range of learning rules to contribute to a variety of behaviors, including cognitive processes. Together, such evidence points to a broad role for cerebellar circuits in generating and testing predictions about movement, reward, and other non-motor operations. However, this expanded view of cerebellar processing also raises many new questions about how such apparent diversity of function arises from a structure with striking homogeneity. Hence, this review will highlight both current evidence for predictive cerebellar circuit function that extends beyond the classical view of error-driven supervised learning, as well as open questions that must be addressed to unify our understanding cerebellar circuit function.

## The cerebellum as a neuronal prediction machine

More than simply a neuronal learning machine, the brain is a prediction machine. Across sensory and motor systems, growing evidence suggests that a key operating principle of the brain is to establish internally generated predictions that can be compared against feedback from the external world in order to guide anticipatory actions and perceptions (Keller and Mrsic-Flogel, 2018).

The cerebellum has long been thought to operate predictively to support motor control and motor learning (Wolpert et al., 1998). As originally proposed by Masao Ito, the cerebellum is hypothesized to utilize a predictive model that anticipates the expected outcome of motor commands in order to refine future movements (Ito, 1970; Ito, 1972). Indeed, decades of research have provided considerable support for this hypothesis (Ohyama et al., 2003), and revealed many of the circuit pathways (Apps and Garwicz, 2005) and mechanisms (Carey, 2011) that allow the cerebellum to predictively modify motor output. However, emerging evidence suggests that the role of the cerebellum in motor control may be more complex than previously appreciated (Medina, 2019). Moreover, it has also become clear that the cerebellum plays a much wider role in brain function than simply refining movements (Buckner, 2013; Leiner et al., 1986; Schmahmann, 1991; Sokolov et al., 2017; Strick et al., 2009). Recently, with advances in modern circuit approaches and the application of more diverse behavioral paradigms in animal models, several studies have shed new light on how cerebellar circuits function across a range of behaviors. In this review, I will highlight some of this progress with the goal of identifying key unifying principles and open questions (Table 1) that are necessary to understand the role of cerebellar processing across diverse motor and non-motor behaviors.

**Table 1.** Open questions.

Can learned climbing fiber activity drive higher order conditioning to establish motor (or other) sequences?
Does climbing fiber activity in reward-based learning paradigms follow the same rules that have been shown for VTA dopamine neurons?
How are reward-related climbing fiber signals generated? Can they be computed locally in the IO, or inherited from upstream brain regions? If inherited, from where?
How are reward-related signals in the granule cells and climbing fibers integrated to mediate learning? How might cerebellar reward-based learning be used by downstream brain regions?
Does the granule cell layer generate sparse representations of sensorimotor input, and how do local synaptic computations establish such representations?
Can climbing fibers generate a graded representation of the magnitude of behavioral errors?
How does cerebellar learning interact with neocortical circuits, and how to do these circuits bi-directionally modulate one another to guide behavior?
How does cerebellar learning modify output to the mesolimbic dopamine system during goal directed behaviors?
How does cerebellar circuit dysfunction modulate neocortical developmental and adult neocortical circuit processing in cognitive disease states such as Autism Spectrum Disorders?
Can cerebellar learning harness different mechanisms and region-specific computations to achieve different goals? Does cerebellar output depend on behavioral or cognitive requirements?

## Classical perspectives on cerebellar learning

**Figure 1.**
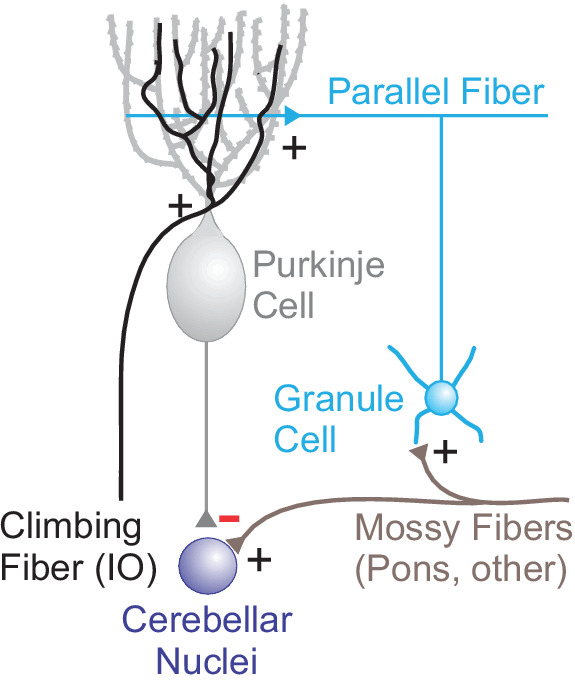
Circuit diagram of cerebellar input and output pathways. Climbing fibers originate in the inferior olive (IO), and excite (plus signs) Purkinje cell dendrites. These inputs serve to instruct heterosynaptic plasticity at synapses from the mossy fiber pathway, an excitatory pathway that originates in the pontine nuclei and elsewhere. The mossy fiber pathway excites granule cells, and terminates in excitatory parallel fiber inputs onto Purkinje cells. Purkinje cells are inhibitory (minus sign), and regulate the activity of output neurons in the cerebellar nuclei.

To refine movements based on the predicted the sensory consequences of action, the cerebellum must solve a credit assignment problem. Specifically, it must attribute deviations between actual and expected sensorimotor feedback to features of movement that occurred in the recent past. Classical models of cerebellar function argue that this problem is solved through a supervised learning rule instructed by inputs to the cerebellar cortex called climbing fibers (CFs, Figure 1; Albus, 1971; Marr, 1969). Supervised learning is characterized by teaching signals that can report whether or not expectations match outcomes (i.e. a yes or no signal). In the cerebellum, CFs are thought to instruct learning by signaling the occurrence of movement errors. These error signals are thought to correct future movement by generating large dendritic calcium spikes (so-called complex spikes, Cspks) in the output neurons of the cerebellar cortex, the Purkinje cells. In turn, Cspks can produce heterosynaptic plasticity on preceding inputs from another pathway, the mossy fiber (MF) to granule cell pathway. Because the MF pathway carries contextual information necessary for learning, such a plasticity rule has long been thought to provide a key substrate for generating cerebellar-dependent supervised motor learning.

This supervised learning rule works well to describe cerebellar activity in the CF and MF pathways during many behaviors, and provides a compelling model to explain how the cerebellum can modify certain movements. However, such supervised learning also requires that CFs have access to a complete menu of erroneous actions in order to provide accurate error-based feedback. This may be practical only for a limited set of behaviors, and particularly for those where the environment can directly indicate what the correct action should have been. In other words, this supervised learning rule is ideal when there is a yoked relationship between stimulus and action, as is common for many well-studied cerebellar-dependent behaviors. Eyelid conditioning provides an illustrative example of a cerebellar-dependent learning task that meets this criterion. In this behavior, animals learn to associate a neutral sensory stimulus with a delayed corneal airpuff that produces a reflexive eyelid closure. By responding via a hardwired neural pathway from the sensory periphery to the same corneal airpuff that produces a reflexive blink, CFs can accurately instruct a predictive eyelid closure according to the principles of supervised learning (Kim and Thompson, 1997; Medina et al., 2000).

In more complex motor behaviors without a fixed stimulus-action relationship, as well as many non-motor behaviors, it has been challenging to understand how such a supervised learning rule could provide an effective means for learning. In particular, in cases where the sensory information necessary for learning has no direct relationship to the movement that requires modification, or when the necessary sensory information is only applicable under a specific behavioral context, it is unclear whether or how CFs could generate such a supervised instructional signal. Indeed, there have been indications from behaviors that meet these criteria that cerebellar supervised learning models are not sufficient to describe CF activity. For example, during arbitrary visuomotor reaching tasks, CF-driven Cspks in Purkinje cells have been shown to reflect predictive signals that are not consistent with motor errors (Kitazawa et al., 1998; Streng et al., 2017). Instead, these studies have shown that Cspks can be predictive of task parameters such as reach destination, upcoming movement kinematics, or future position errors. Even during eyelid conditioning, recent evidence suggests that the cerebellum may be able to harness a wider range of distinct learning rules to modify behavior.

## Predictive coding in climbing fibers

**Figure 2.**
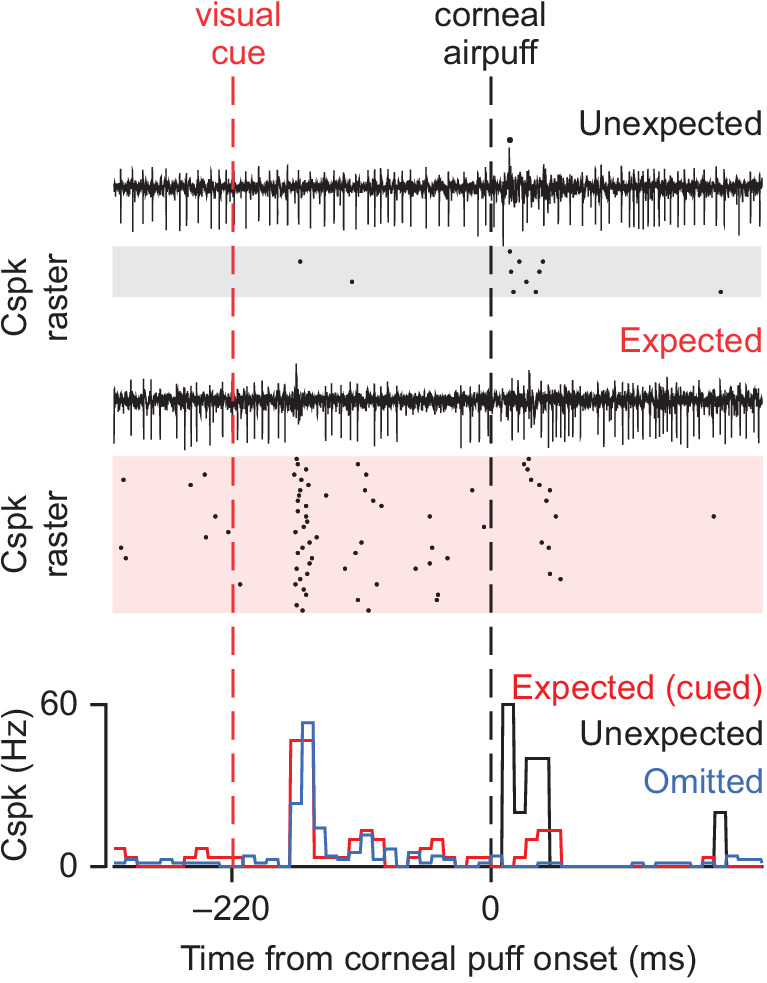
CF input to Purkinje cells obeys the principles of TD learning. CF-driven Cspks, recorded here after learning, are more probable in response to a US (corneal airpuff) that is unexpected (black) than when the same stimulus is expected (red). Cspks also follow the conditioned stimulus after learning, and are reduced below baseline levels when the expected US does not occur.

In a landmark study, Ohmae and Medina provided a new blueprint for how cerebellar circuits may operate to enable learning beyond a supervised context (Ohmae and Medina, 2015). By recording from the cerebella of awake mice locomoting on a treadmill during eyelid conditioning, the authors demonstrated that CFs could provide a different type of learning signal; namely, one that meets the criteria described by temporal-difference (TD) models of reinforcement learning. In a TD learning framework, teaching signals exhibit key properties that change both what and how a system can learn relative to a supervised learning framework (Sutton and Barto, 1998). Specifically, in TD learning, teaching signals are scalar, and vary according to current expectations. Indeed, the authors found that CF activity met this criterion, as Cspks were more probable in response to an unconditioned stimulus (US; i.e. corneal airpuff) that was unexpected than when the same stimulus was expected (Figure 2). Importantly, the probability of Cspks on unexpected US trials was higher than for expected US trials where no conditioned response (CR) was generated, suggesting that differences in sensory encoding of the US were not responsible for the differences in Cspk probability. These findings hence contradict what would be predicted by a supervised learning model, which would instead suggest the same Cspk probability on any trial type without a predictive eyelid closure to block the aversive corneal airpuff.

Another key feature of TD learning models is that teaching signals are modulated by experience to represent higher-order reinforcing stimuli. Again, CF activity met this criterion, as Cspks emerged in response to the conditioned stimulus (CS; i.e. an LED that reliably preceded the airpuff) after learning (Figure 2) (See also ten Brinke et al., 2015). No such CS-related Cspk responses would be predicted in supervised learning models. In TD models however, these properties of teaching signals (scalar responses based on expectation and learned responses to higher-order reinforcing stimuli) allow the system to learn by trial-and-error exploration without the need for prior knowledge about a correct outcome (e.g. the final, correctly-executed learned movement). Such properties may be ideal to enable a cerebellar contribution to learning across a range of motor and non-motor behaviors, and in particular behaviors where learning is guided by predictions about upcoming reward.

**Figure 3.**
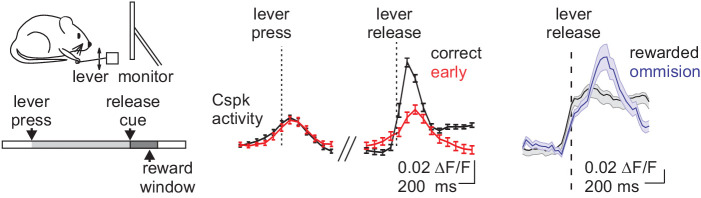
Cspk activity reflects predictions about reward delivery. *Left,* mice were trained to press and release a lever as instructed by a visual cue in order to receive reward. *Middle,* Cspk activity was greatest for lever releases that predicted reward delivery. *Right,* Cspk activity was enhanced when expected reward was not delivered.

The neural seat of reward-guided reinforcement learning has historically been considered to be the striatum, where projections from VTA dopamine neurons largely obey the principles of TD models to instruct synaptic plasticity and learning about reward-predictive events (Glimcher, 2011). Recent work has provided compelling evidence that the cerebellum may also contribute to reward-based reinforcement learning (Heffley and Hull, 2019; Heffley et al., 2018; Kostadinov et al., 2019; Larry et al., 2019). For example, two studies using calcium imaging to visualize Cspk activity in awake mice during operant learning tasks have now demonstrated that CFs can exhibit responses that are consistent with reward-based reinforcement learning signals (Heffley et al., 2018; Kostadinov et al., 2019; Figure 3). In each of these studies, mice were trained to execute a voluntary action cued by a neutral sensory stimulus in order to receive reward. In these behaviors, both groups found that Cspks can reflect actions or events that predicted upcoming reward in a scalar manner that was proportional to reward expectation. In addition, these studies found that Cspks also report violated expectations by signaling when an expected reward is not delivered (Figure 3). These results are not only consistent with reinforcement learning, but directly oppose the motor error hypothesis of CF activity. Specifically, because Cspk activity was generated in response to actions or events that accurately predicted upcoming reward (Heffley et al., 2018), this activity necessarily occurred when animals correctly executed movements rather than when movement was mis-executed.

**Figure 4.**
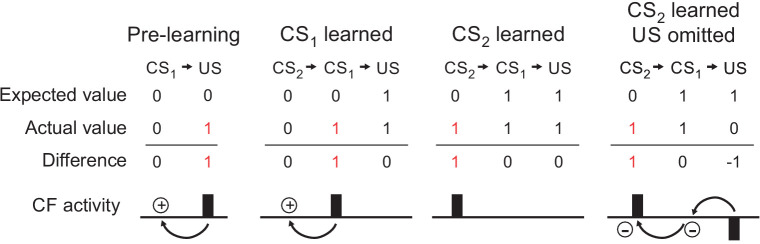
Schematic of CF activity in a TD learning framework. Before learning, CFs are driven by an unexpected US. After learning, CFs are driven by a CS that accurately predicts the US (CS_1_). If a new CS occurs earlier in time (CS_2_), CF activity can then be driven by this higher order stimulus. Finally, if the expected US is omitted, CF activity is reduced (negative prediction error), serving to extinguish associations with the no longer appropriate CS_1_ and CS_2_.

It initially appears paradoxical that CF learning signals should occur in response to movements that result in reward, and thus do not immediately require modification. However, it is important to note that the reward-predictive Cspk responses associated with movement in these studies occurred in the learned condition, when the expectation that a specific movement or event would result in reward had already been established. At this timepoint, reinforcement learning principles suggest that Cspks should be used to drive second order conditioning (Figure 4). In other words, if a new stimulus and/or action emerged that reliably provided an even earlier predictor of reward, these CF-driven signals would be ideally situated to drive a higher order learned association to that new event.

Such a mechanism could enable the association of multiple actions in order to establish a fully coordinated movement. To date, cerebellar-dependent sequence learning has been demonstrated only when movement feedback signals are reinforced by exogenously stimulated CF activity (Khilkevich et al., 2018). These data provide an exciting proof of principle for how cerebellar learning can establish compound movements. However, it remains to be tested whether learned CF responses to conditioned stimuli can in fact support higher order conditioning and/or motor sequence learning. It also remains plausible that learned CF responses to conditioned stimuli can allow further modification of movement by other means, for example by directly modulating the activity of CbN neurons (Ten Brinke et al., 2019; Ten Brinke et al., 2017), or may serve a different purpose altogether by enabling cerebellar output to downstream brain regions. Testing such predictions will be crucial for understanding how learned, conditioned stimulus-driven CF signals are harnessed for modifying behavior.

Interestingly, while the Cspk signals reported in both Heffley et al. and Kostadinov et al. are consistent with reinforcement learning, they contrast in key ways with the Cspk patterns described by Ohmae and Medina. In particular, neither Heffley et al. nor Kostadinov et al. observed a decrease in Cspk activity when expected reward was not delivered. Instead, both studies reported elevated Cspk activity in response to defied expectations. Such responses are consistent with unsigned prediction errors, but not with the types of signed prediction errors typically associated with TD learning (Figure 4). The decreases in Cspk activity demonstrated by Ohmae et al. are ideal to promote extinction learning during eyeblink conditioning when the aversive conditioned stimulus is absent (Medina et al., 2002), and may therefore also be a hallmark of behaviors with fixed stimulus-action relationships. In contrast, the increased Cspk responses shown by Heffley et al. and Kostadinov et al. may promote exploration and new associations during behavior that requires flexible stimulus-action relationships. Hence, despite the commonality of learned Cspk responses to higher-order reinforcing stimuli across studies, it remains to be determined how specific task requirements and location within the cerebellum determine the CF response to violated expectations.

## Origins of climbing fiber reinforcement learning signals

How might CF reinforcement signals be generated? The axons that form CFs originate from neurons in the inferior olive (IO). In many cerebellar-dependent behaviors, input to the IO that drives CFs and learning comes directly from the sensory periphery. For example, in eyelid conditioning, the corneal airpuff used as a US is transmitted to the IO via the trigeminal ganglion (Kubo et al., 2018; Swenson and Castro, 1983; Van Ham and Yeo, 1992), reliably triggering CF input to the cerebellum. This ‘hardwired’ pathway allows CFs to respond with high fidelity in a manner consistent with supervised learning based on the causal association between stimulus and movement. However, the IO also receives indirect input from both cortical and subcortical brain regions that may allow it to generate more complex teaching signals than can be produced by salient sensory input from the environment (Ten Brinke et al., 2019).

One such key source of input to the IO is the mesodiencephalic junction (MDJ) (De Zeeuw et al., 1998). This region of the midbrain is composed of multiple nuclei, some of which integrate cerebellar CbN output and project to either downstream neurons in the spinal cord (Keifer and Houk, 1994) or the IO (Onodera, 1984). Because cerebellar learning has been shown to produce new mossy fiber collaterals in the CbN (Boele et al., 2013; Kleim et al., 2002; Lee et al., 2015; Weeks et al., 2007), it has been speculated that such collaterals may convey information about learned conditioned stimuli to the MDJ, and in turn to the IO (Ten Brinke et al., 2019; Ten Brinke et al., 2017). In this model, mossy fiber pathways carrying learned information about conditioned stimuli could be indirectly translated to the IO via these new collaterals, producing instructive reinforcement learning signals in CFs. Notably, such an extended polysynaptic pathway may partly explain why CF signals related to conditioned stimuli tend to have longer latencies than the US-related CF signals that are translated more directly from the sensory periphery.

The MDJ also integrates descending input from sources that include cortical pyramidal tract neurons (Veazey and Severin, 1982). Such input likely allows the IO to represent higher order cortical computations, and is therefore a good candidate to transmit the types of reward prediction and TD-learning signals that have been shown recently.

The IO also receives inhibitory input, and contains some local interneurons, raising the possibility that local computations may also enrich the repertoire of CF responses (De Zeeuw et al., 1998). For example, the IO receives inhibitory input from the cerebellar nuclei (CbN) (Bengtsson and Hesslow, 2006). These inhibitory projections participate in extinction learning (Medina et al., 2002), but could also play a role in gating CF activity or sculpting responses to incoming excitation. For example, inhibitory inputs could decouple stimulus-action relationships resulting from peripheral IO inputs, enabling other pathways to dominate when learning requires information from other sources. Indeed, multiple studies have found that CF activity exhibits context dependence, and that sensory responses can be actively suppressed under certain behavioral conditions (Apps, 1999; Apps and Lee, 1999; Gellman et al., 1985; Horn et al., 1996; Kim et al., 1987). Whether inhibitory CbN projections play a role in such context-dependent IO suppression remains unclear. More broadly, it has remained challenging to establish any clear predictions about the influence of discrete pathways in generating CF learning signals, as there remains an incomplete description of inputs to the IO, as well as a limited understanding of when different pathways are active and how the IO integrates input to generate CF responses *in vivo*. Thus, a key step in understanding how CFs can produce complex teaching signals such as those necessary for reinforcement learning will be to establish a more detailed map of input to the IO, and to measure the behavioral contexts under which specific input pathways recruit CF activity.

## Predictive coding in granule cells

To mediate cerebellar learning, the teaching signals carried by CFs are thought to instruct heterosynaptic plasticity at excitatory synapses from granule cells onto Purkinje cells. Thus, the information carried by granule cells crucially determines what the cerebellum can learn.

Classical models of the granule cell layer suggest that it serves a key role in pattern separation by expanding, sparsifying and decorrelating cerebellar input in order to maximize the number of unique representations that can be learned by Purkinje cells (Albus, 1971; Marr, 1969). Indeed, the sheer number of granule cells makes sparse coding models appealing, as these neurons are by far the most numerous in the brain, and significantly outnumber their presynaptic mossy fiber inputs (Eccles et al., 1967). Their activity has also been thought to be kept sparse by synaptic inhibition, which reduces the threshold and gain of granule cell responses to incoming mossy fiber input (Chadderton et al., 2004; Duguid et al., 2012; Mitchell and Silver, 2003). Beyond sparsity, the idea that the granule cell layer can decorrelate inputs has also been supported by observations that individual granule cells can pool inputs from different sources (Huang et al., 2013) and with different synaptic strengths (Chabrol et al., 2015).

In contrast with classic models, recent work has challenged the idea that granule cells generate sparse representations, and shown that they can instead exhibit dense and redundant responses during several behaviors (Giovannucci et al., 2017; Knogler et al., 2017; Ozden et al., 2012; Sylvester et al., 2017). Such results are surprising, and may suggest that it is necessary to rethink how the cerebellum forms unique sensorimotor associations. However, an alternative possibility is that some aspects of the original Marr-Albus models require revision. For example, pattern separation need not require sparse coding (Cayco-Gajic and Silver, 2019). In particular, as argued by Cayco-Gajic and Silver, pattern separation can be achieved without sparse coding if inputs are still expanded and decorrelated, allowing dense granule cell responses to effectively discriminate complex, high-dimensional inputs. It should also be noted, however, that the dense granule cell responses measured thus far have been largely observed during complex behaviors. In these cases, there are likely to be many sensorimotor patterns represented simultaneously. Thus, whether the cerebellar granule cell layer acts to sparsify discrete sensorimotor inputs, and what mechanisms the granule cell layer uses to generate unique representations, remains an open question.

Apart from how the granule cell layer processes incoming input, recent work has also extended our view of what the granule cells can represent. Previous work across many cerebellar-dependent learning paradigms had revealed considerable evidence that the granule cells encode the predictive context necessary for motor learning (Raymond and Medina, 2018; Sawtell, 2017). For example, in associative motor learning tasks, granule cells carry information about the predictive CS (Steinmetz et al., 1989), allowing Purkinje cells to develop learned responses to these inputs (Halverson et al., 2015; Hesslow and Ivarsson, 1994). Likewise, for adaptation learning paradigms such as vestibulo-ocular gain learning, the granule cells receive copies of learned motor commands, or so-called efference copies, that can provide a basis for predictive learning (Lisberger and Fuchs, 1978a; Lisberger and Fuchs, 1978b). Indeed, recent calcium imaging data further supports the idea that granule cells can represent efference copy information (Giovannucci et al., 2017). Using calcium imaging to measure the responses of granule cells across eyelid conditioning, Giovannucci et al. revealed learned representations of the conditioned eyelid closure that can match, or even precede the eyelid movement after learning.

To establish predictive contextual representations, granule cells appear to employ population codes that take advantage of input that is tuned to specific stimulus or kinematic parameters. For example, elegant *in vivo* recordings from rodents have revealed that granule cells can be narrowly tuned to movement features such as whisker position (Chen et al., 2017). By linearly encoding such features according to the properties of synaptic transmission from mossy fibers (Arenz et al., 2008; Duguid et al., 2012; Powell et al., 2015; Rancz et al., 2007), granule cells can effectively relay a population code to downstream Purkinje cells that faithfully represents precise features of upcoming movement kinematics (Chen et al., 2016).

**Figure 5.**
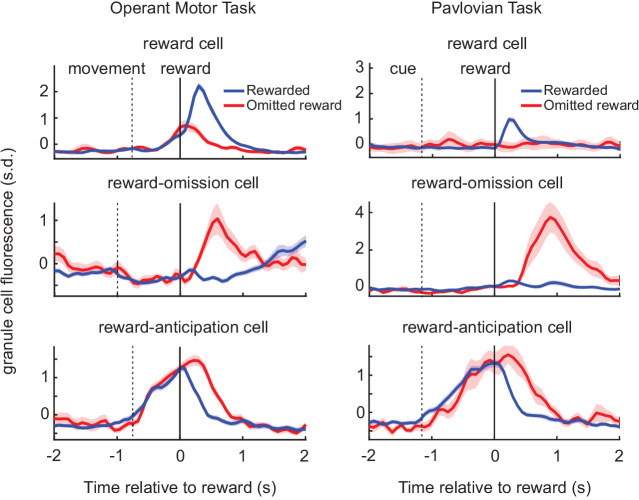
Granule cells develop reward-predictive activity in both operant and Pavlovian conditioning tasks. *Left,* population level calcium imaging in mice reveals distinct populations of granule cells that represent reward delivery (top), reward omission (middle), and the anticipation of reward delivery (bottom) after learning in an operant forelimb task. *Right*, the same categories of responses arise following learning in a Pavlovian task where a neutral cue (CS) is associated with reward.

Surprisingly, however, predictive coding in granule cells now appears to extend beyond the motor domain, and can also reflect cognitive predictions. Using both operant and Pavlovian tasks guided by reward reinforcement, Wagner and colleagues used calcium imaging to demonstrate that granule cells can develop non-motor predictions (Wagner et al., 2017; Figure 5). Specifically, this study revealed that granule cells can develop learned representations of both actions and stimuli that predict upcoming reward, with as many as 25% of the total recorded cells responding to reward, reward omission, or reward anticipation. That such predictions need not be exclusively related to movements is particularly exciting, and lends support to the idea that granule cells can provide a substrate for cerebellar learning that is not exclusive to motor control. If so, such predictions may be used by downstream targets in the neocortex and elsewhere for a variety of computations.

## Predictive coding in purkinje cells

How do the various sensory, TD, and reward-prediction signals carried by climbing fibers combine with contextual information in the granule cells at the level of Purkinje cells to allow the cerebellum to evaluate and update its model of the world? Several elegant studies have now begun to reveal that the sodium-based action potentials of Purkinje cells (so-called ‘simple spikes’) can establish complex kinematic predictions about movement across diverse behaviors (Brown and Raman, 2018; Chen et al., 2016; Ebner and Pasalar, 2008; Herzfeld et al., 2015; Pasalar et al., 2006). Such data support the notion that the cerebellum can harnesses a type of predictive computation often termed a ‘forward model’. A forward model can be most simply conceptualized as an estimation of the immediate future based on a copy of the current motor command and current sensory information. A key virtue of such predictions is that they can be used as a more rapid substitute for external feedback (e.g. the sensory consequences of movement) to enable anticipatory actions. Such forward model predictions can also be compared with subsequent sensorimotor feedback to assess differences between expectation and outcome. When there is a mismatch, termed a ‘sensory prediction error’, the forward model can be updated via learning mechanisms (e.g. synaptic plasticity) in accordance with current conditions.

While CF input to Purkinje cells is typically considered to be the teaching signal necessary to update cerebellar forward models, there is also evidence that the granule cell pathway may carry feedback error signals (Popa and Ebner, 2018). Such error signals have been observed in the simple spiking of Purkinje cells in a manner that is independent of Cspks (Popa et al., 2012; Popa et al., 2017; Streng et al., 2018). These data imply that Purkinje cells can carry the necessary information for updating a cerebellar forward model without CFs in some cases. These results are also consistent with the finding that CF activity is not required for some forms of cerebellar learning (Ke et al., 2009; Kimpo et al., 2014), and may also support the idea that Cspks can serve alternate roles in some behaviors (Streng et al., 2017).

**Figure 6.**
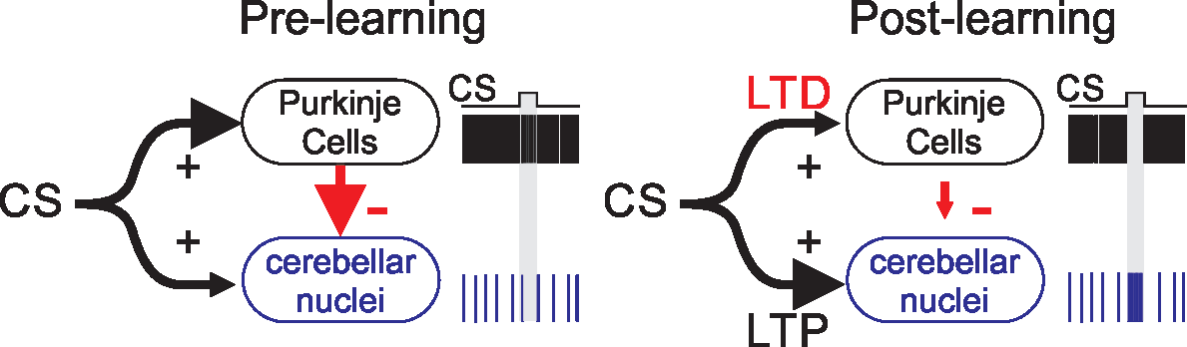
LTD is thought to provide a key mechanism for reducing Purkinje cell output to enable learning. Parallel fibers (Figure 1) in the mossy fiber pathway carrying CS input are thought to be depressed when paired with CF input to Purkinje cells. This enables a well-timed reduction in Purkinje cell inhibition of CbN cells. LTP of mossy fiber input to CbN cells may also facilitate learning (Pugh and Raman, 2006).

In cases where CF activity is strongly linked to learning, the primary model suggests that these signals serve to instruct long-term synaptic depression (LTD) of granule cell synapses onto Purkinje cells (Albus, 1971; Ito, 1972). This mechanism is appealing because Purkinje cells are inhibitory, and exhibit high convergence onto target neurons in the CbN that form the output of the cerebellum (Person and Raman, 2012). Thus, predictive cerebellar output from CbN neurons would be greatly facilitated by an appropriately timed disinhibition that could be achieved by reducing the simple spiking of Purkinje cells (Heiney et al., 2014; Figure 6).

**Figure 7.**
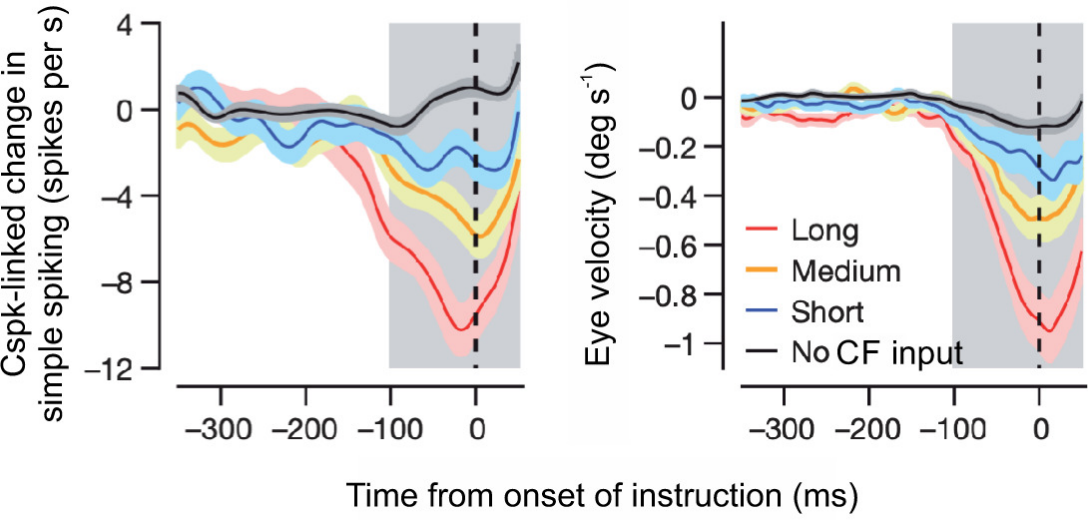
Cspk activity is correlated with both the depression of Purkinje cell simple spiking and learning on a trial-by-trial basis. *Left,* Cspks on the preceding trial lead to a depression of Purkinje cell simple spiking on the next trial that is proportional to the duration of the Cspk. *Right*, trial over trial changes in eye velocity obey the same relationship to Cspk duration as the depression in simple spiking.

Indeed, while current evidence suggests a role for plasticity of various types and at multiple sites in the cerebellar circuit (Boyden et al., 2004; Carey and Lisberger, 2002; Gao et al., 2012), there remains considerable evidence that CF-driven LTD, or at least CF-driven reductions in PC simple spiking, is involved with many forms of cerebellar learning (Ito et al., 2014). In particular, studies of learned eye movements have provided the most direct evidence to date for a causal link between Cspk-driven reductions in Purkinje cell simple spiking and learning (Herzfeld et al., 2018; Kimpo et al., 2014; Medina and Lisberger, 2008; Yang and Lisberger, 2014; Figure 7). This work has shown that Cspks are highly correlated with a depression of PC simple spiking and learning on a single trial basis. Moreover, these studies have made a key link between the duration of Cspks and learning, revealing that these signals are graded, likely due to graded presynaptic CF activity (Gaffield et al., 2019), in a manner that scales with the amount of single trial learning. These findings suggest the possibility that the duration of Cspks may be also be determined by behavior, with larger errors leading to longer duration or more probable CF input (Najafi et al., 2014; Najafi and Medina, 2013). Thus, further exploring the relationship between behavioral variability (e.g. error magnitude), CF activity, Cspks, and the depression of PC simple spiking will be crucial to understanding the links between learning and its underlying mechanisms. Moreover, while considerable evidence suggests that the depression of Purkinje cell simple spiking provides at least part of the necessary substrate for predictive cerebellar output, it remains necessary to make direct links between learning and LTD. Despite key efforts in this direction (Schonewille et al., 2011; Yamaguchi et al., 2016), conclusive tests of how LTD at PC synapses contributes to learning will require manipulations that are not only cell-type specific, but also temporally specific in order to overcome the complications of circuit compensation that are inherent to chronic genetic strategies.

## Prediction signals in the cerebellar nuclei

At the level of the CbN, direct evidence to illustrate predictive cerebellar output has been somewhat limited. Clear examples had previously existed only for a small number motor behaviors with similar learning requirements. For example, during eyelid conditioning, CbN neurons become active to the conditioned stimulus to enable a predictive eyelid closure by activating downstream motor neurons (McCormick and Thompson, 1984). Until recently, however, there had been little evidence to support the idea of predictive cerebellar output during behaviors other than those that involve simple stimulus-action associations, and no clear neural data showing how cerebellar output might vary according to expectations associated with different sensory inputs.

**Figure 8.**
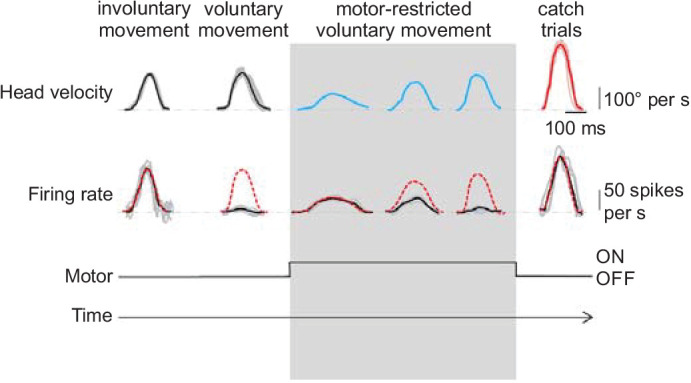
CbN neuron spiking is proportional to mismatch between actual and predicted head velocity. *Top,* head velocity traces for a monkey making voluntary and involuntary (imposed via rotational turntable) head movements. When force is applied from an external motor (gray interval), the monkey must slowly adapt its head movement to account for the oppositional force and recover normal head velocity. Catch trials are interleaved where no motor-restriction is applied. *Middle*, a representative CbN neuron fires only when head movement differs from expectation, and its firing rate is directly proportional to the difference between actual and expected head movement across time.

By recording from the CbN of awake behaving primates, Brooks and colleagues showed that these neurons can dynamically track the difference between expected motor output and sensory feedback, consistent with the computation of sensory prediction error during voluntary head movements (Brooks et al., 2015). In particular, they found that CbN neurons responded to mismatches between expected and actual head movements on a trial-by-trial basis (Figure 8). Moreover, these prediction-dependent CbN responses were continuously updated with new learning. These results strongly suggest that cerebellar output can reflect the computation of a prediction error that results from comparing an internal model of the sensory consequences of active head movement with actual sensory feedback. Such data are thus consistent with the forward model hypothesis, and imply that the internal model’s prediction lies upstream of the CbN neurons, perhaps instantiated by the spiking of Purkinje cells. Notably, CbN neurons in this study were modulated in the same direction regardless of the direction of sensorimotor mismatch. Specifically, CbN neurons elevated their firing regardless of whether head movement was unexpectedly restricted, or unexpectedly released from restriction during extinction learning. Such unidirectional signaling of mismatch is likely appropriate to drive stabilizing vestibluo-spinal reflexes (Roy and Cullen, 2001; Roy and Cullen, 2004) and to ensure stable perception that accounts for self-motion (Dale and Cullen, 2019). It has remained challenging, however, to establish such causal relationships between CbN activity and behavior, as tools to selectively manipulate CbN neurons in a manner that accounts for ongoing behavior have not been available until recently.

To begin addressing the casual relationship between CbN activity and behavior, a recent study has used closed-loop optogenetic manipulations to alter the firing of CbN neurons during movement (Becker and Person, 2019). By both activating and inhibiting CbN neurons at different timepoints during skilled reaching, this study revealed that the activity of these cells contributes to predictively controlling the endpoint of reaches in real time. Specifically, the authors found that CbN output contributed unidirectionally to limb movement, adding velocity toward the body regardless of limb movement direction. These data suggest that the cerebellum can contribute at least part of the motor command necessary to regulate ongoing movements on a millisecond timescale, calibrating behavior even during steady state performance after learning has occurred. Notably, such CbN activity that persists after learning differs considerably from the patterns measured by Brooks and colleagues, where CbN output was abolished after learning (provided movement proceeded as expected). In absence of a clear distinction between the features of learning across studies such as these, it seems that we are only at the early stages of understanding how cerebellar output 1) contributes to motor control across diverse behaviors and learning paradigms, and 2) conforms to predictions about the implementation of cerebellar forward models. Thus, while forward model explanations have gained considerable support from behavioral studies in humans (Izawa et al., 2012; Morton and Bastian, 2006) and animals (Machado et al., 2015; Pasalar et al., 2006), it will be necessary to further probe the links between theory and newly emerging datasets. For example, it will be crucial to determine whether and how behavioral demands, cerebellar region, and downstream target area dictate the mode of cerebellar output. Such efforts will be challenged by the widening range of brain regions and behaviors and that the cerebellum contributes to, including those that are now recognized to involve complex cortical computations.

## Cerebellar influence on neocortical predictive coding

**Figure 9.**
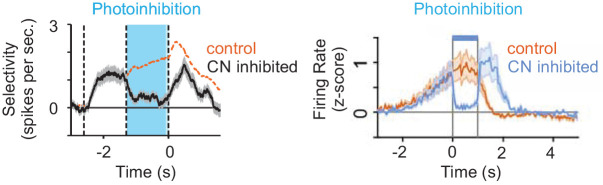
Inhibiting cerebellar CbN output abolishes ramping activity in ALM. *Left,* inhibition of the CbN (fastigial) prevents ALM ramping in a whisker-based sensory discrimination task. *Right,* inhibition of the CbN (dentate) prevents ALM ramping in a virtual reality conditioning task. Modified from Gao et al. (2018) (Left) and Chabrol et al. (2019) (Right) with permission from N Li, K Svoboda, CI DeZeeuw (left) and T Mrsic-Flogel (right).

Recently, studies focused on neocortical areas have suggested a cerebellar influence on downstream targets that is at least reminiscent of forward model predictions (Chabrol et al., 2019; Gao et al., 2018). In addition to descending rubrospinal pathways, the cerebellum is heavily connected to the neocortex disynaptically via the thalamus, including pathways to sensory, motor and premotor cortical areas (Kelly and Strick, 2003; Proville et al., 2014). To test how cerebellar output modulates motor-related cortical processing, Gao et al. examined how cerebellar output affects activity in the anterior lateral motor cortex (ALM). This neocortical region is involved in motor planning, and exhibits persistent ramping activity prior to movement that is necessary for accurate motor performance (Guo et al., 2014; Li et al., 2016). Remarkably, by optogenetically inhibiting cerebellar CbN neurons, Gao et al. found that cerebellar output was necessary for ramping activity in ALM during a motor discrimination task (Figure 9). Moreover, disrupting cerebellar CbN output impaired motor-based decisions by introducing a motor bias, but did not disrupt motor output per se. These results argue for a key role of cerebellar output in motor planning, and perhaps more broadly in predictive cerebral cortical computations across many domains.

CbN output to ALM may also reflect the expected outcome of motor plans. Similar to the results of Gao et al., Chabrol et al. found that cerebellar CbN output was necessary for ramping in ALM (Gao et al., 2018; Chabrol et al., 2019; Figure 9). However, this study focused on a more lateral region of the CbN, and revealed that the impact of cerebellar output on ALM was restricted to a behavioral context where a visual cue predicted the timing of upcoming reward. This result is in line both with evidence that the cerebellum establishes temporal predictions (Mauk et al., 2000), and that the lateral cerebellum may be especially involved in temporal predictions related to reward (Heffley and Hull, 2019; Heffley et al., 2018; Kostadinov et al., 2019).

Together, these ALM results are consistent with studies in non-human primates showing that neurons in the lateral CbN can exhibit predictive ramping responses during a delay period prior to movement (Ashmore and Sommer, 2013; Ohmae et al., 2017). While such CbN ramping responses have been implicated in action timing, it is reasonable to extrapolate that temporal signals of this type could be used by the neocortex for a variety of timing computations. Similar to delay-interval ramping, CbN neurons have also been shown to predictively represent the timing of periodic stimuli with oscillatory responses that increase prior to each stimulus presentation. Such responses appear to reflect temporal predictions rather than expectations about other stimulus features, as the same neurons preferentially signaled when temporal periodicity was unexpectedly violated (Kameda et al., 2019; Ohmae et al., 2013). Thus, it appears that the cerebellum can transmit various timing predictions to the neocortex, an idea that is borne out by the impairment of movement timing when the cerebellar thalamocortical pathway is selectively disrupted (Nashef et al., 2019). Notably, however, the cerebellar thalamocortical pathway projecting to primary motor cortex appears not to signal via anticipatory pre-movement ramping. Rather, motor cortical responses driven by this pathway exhibit transient excitation followed by a long-lasting recruitment of cortical inhibition (Nashef et al., 2018). While it is unclear how the CbN neurons projecting to the motor thalamocortical pathway fired in that study, the data are at least suggestive that predictive ramping activity is not the sole mode of cerebellar output to motor cortical areas during timing tasks.

Overall, these studies may point toward a forward-model explanation for how cerebellar output affects neocortical areas, as in each case the cerebellum appears to signal temporally-specific predictions about behavioral outcomes related to the function of these areas. However, much work remains to rigorously test this hypothesis, and will require a more complete understanding of the cerebellar computations involved in neocortical processing, as well as a clear description of how signals from discrete cerebellar output pathways act to sculpt neocortical activity.

Because the neocortex also feeds back to the same cortical targeting regions of cerebellum via the pons in a closed-loop manner (Kelly and Strick, 2003; Proville et al., 2014; Strick et al., 2009), it is likely that the predictions made by cerebellum are subject to ongoing refinement by descending feedback. Indeed, recent work has demonstrated that activity in the cerebellar granule cell layer becomes more correlated with neocortical activity as a function of learning in a goal directed task (Wagner et al., 2019). Thus, it will also be crucial to understand how these circuits bi-directionally modulate one another in a coherent manner to alter behavior (Siegel and Mauk, 2013).

## Expanding roles for the cerebellum in behavior

Over the last several decades, there has been a growing appreciation that the cerebellum contributes to a wide range of non-motor processes (Schmahmann, 1991), including cognition (Kim et al., 1994), social processing (Van Overwalle et al., 2014), aggression (Reis et al., 1973) and emotion (Schmahmann and Caplan, 2006). In agreement with these findings, humans with cerebellar damage or disease often exhibit non-motor conditions, including autism spectrum disorders (Wang et al., 2014), deficits of language processing and vocal learning (Ackermann, 2008), schizophrenia (Mothersill et al., 2016) and temporal processing impairments (Ivry and Spencer, 2004). Such findings in humans provide a crucial starting place for identifying the specific cerebellar circuit pathways that contribute to non-motor behaviors, and for establishing testable hypotheses about how such circuits operate.

**Figure 10.**
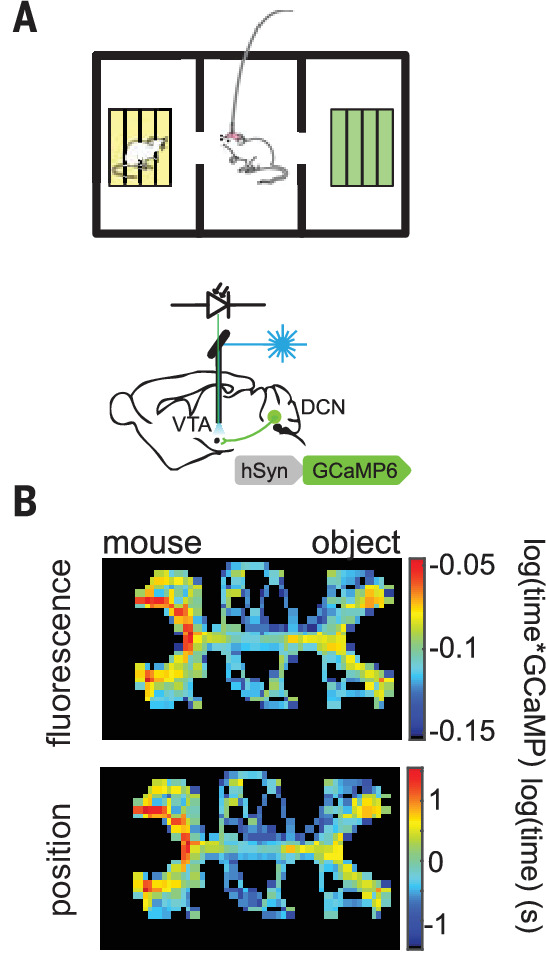
CbN projections to the VTA signal during a social behavioral context. *Top,* behavioral chamber where a test mouse can explore either a novel object (green area) or novel animal (yellow area). Fiber photometry was used to measure the activity of VTA projecting CbN neurons. *Bottom,* VTA projecting CbN neurons are preferentially active when the test mouse explores the novel animal.

Indeed, motivated by such findings, Strick and colleagues have used transynaptic rabies tracing methods to elucidate several discrete pathways from the cerebellum to brain regions other than the motor cortex (Strick et al., 2009), including pathways to the basal ganglia via the thalamus (Bostan and Strick, 2018; Hoshi et al., 2005). Such connections support the idea that the cerebellum can participate in motivation and reward-driven behaviors. In agreement with this hypothesis, a key recent study has shown that the lateral cerebellum also has a direct, monosynaptic connection to the ventral tegmental area (VTA) in mice (Carta et al., 2019). Beyond demonstrating a functional connection from cerebellum to VTA, this study also provided evidence that this same pathway can positively modulate reward-driven behaviors, and is endogenously activated under social conditions (Figure 10). These data strongly suggest that the cerebellum contains, or has the ability to learn, information about rewarding stimuli. Such a model fits well with recent work showing reward-predictive Cspks across the lateral cerebellum during a classical conditioning task similar to those commonly used to study reward processing in striatal circuits (Heffley and Hull, 2019). If combined with contextual information from the mossy fiber pathway, such reward-predictive Cspks could effectively instruct cerebellar output to the VTA in response to reward-associated stimuli or actions. To evaluate such possibilities, a key next step will be to test how cerebellar learning modifies output to the mesolimbic dopamine system during goal-directed behaviors.

Following similar guidance, another recent study has identified a circuit pathway that provides a link between cerebellar output and vocal learning (Pidoux et al., 2018). Previous functional imaging work in humans has demonstrated cerebellar activation during both external (spoken) and internal (unspoken) speech (Ackermann, 2008). Moreover, children with cerebellar dysfunction can exhibit significantly delayed vocal learning (Ziegler and Ackermann, 2017). Based on these findings, Pidoux and colleagues used the songbird to identify a discrete circuit connecting the lateral cerebellum to a part of the avian basal ganglia required for song learning, and revealed that this circuit plays a preferential role in learned vocal timing (Pidoux et al., 2018). By identifying such a pathway, this study opens the door for targeted manipulations capable of revealing the relationship between cerebellar learning and speech production in vocal learning species.

Finally, another vital insight from human studies has come from the many observations that neurodevelopmental disorders, and particularly autism spectrum disorders (ASDs), strongly correlate with cerebellar damage during birth and mutations in cerebellar genes (Sathyanesan et al., 2019; Wang et al., 2014). These insights have led to multiple studies in animal models showing that mutations in genes associated with ASDs can alter cerebellar circuit function (Baudouin et al., 2012; Behesti et al., 2018; Piochon et al., 2014; Tsai et al., 2012), and can also produce ASD-like phenotypes (Tsai et al., 2012). More recently, key studies have begun to reveal pathways from the cerebellum to frontal cortical regions that may mediate such deficits (Badura et al., 2018; Stoodley et al., 2017). Whether and how cerebellar dysfunction alters developmental processes in downstream brain regions, disorganizes activity in mature downstream circuits, or both, will be crucial to understanding the cerebellar role in disorders with significant cognitive components.

Together, such studies have highlighted the importance of using information gleaned from human studies of cerebellar activity and disease states to guide circuit based interrogations in animal models. However, as new work continues to emerge suggesting cerebellar contributions to a wide range of motor and non-motor behaviors, a key challenge will be to identify what common principles may link the role of cerebellar predictions across behaviors, or what properties of cerebellar computation may be unique to individual behaviors.

## Conclusions and open questions

A meaningful understanding of how cerebellar circuits operate predictively must span multiple levels of inquiry, merging mechanistic insights at the cellular and synaptic level with functional explanations of how cerebellar computation modifies behavior. Thus, in considering a way forward, it can be useful to organize the question of what experiments are necessary according to a conceptual framework. As is frequently remembered (Diedrichsen et al., 2019; Keller and Mrsic-Flogel, 2018), Marr (1982) three levels of analysis provide such a framework. Termed ‘computational’, ‘algorithmic’, and ‘implementational’, these levels refer respectively to the problem that needs to be solved, the computation necessary to solve the problem, and the hardware tasked with implementing the computation.

At the level of computation, it would initially seem that the cerebellum is tasked with solving multiple problems, as it clearly contributes to both motor control and diverse cognitive processes. However, this information alone does not resolve the question of what specific problem the cerebellum is tasked with, as its discrete contribution to such diverse behaviors remains opaque. Indeed, the question of whether the cerebellum performs a so-called ‘uniform transform’ (Schmahmann, 1996) or exhibits ‘multiple functionality’ (Diedrichsen et al., 2019) has recently been addressed elsewhere (Diedrichsen et al., 2019). From the perspective of a circuit-based analysis of cerebellar computation, such distinctions cannot be made exclusively by evaluating changes in behavior in cases of cerebellar damage or disease in humans, or following manipulations that impair cerebellar output in animal models. Rather, to determine what computation(s) the cerebellum mediates, and whether they are behavior-specific, it will be necessary to measure 1) exactly what signals the cerebellum sends to different brain regions, and 2) how these signals combine with other inputs to modulate local processing. For example, if all cerebellar output signals are consistent with forward model predictions, they should reflect temporal or state estimations that anticipate the consequences of actions or thoughts in a manner that is relevant to the processing goals of the targeted brain region. However, cerebellar computation may not be restricted to the generation of a forward model, as other types of predictive models have also been proposed for cerebellar computation (Kawato, 1999). Moreover, there are reasons to suspect that cerebellar computation could vary across different phases of learning, allowing the cerebellum to implement different predictive models as synaptic plasticity mechanisms are engaged at different points in the circuit (Medina, 2011). Thus, because there remain several viable possibilities to describe cerebellar computation, and no defined expectation for what state estimates or command signals are necessary for behaviors whose read-out is less straightforward than movement, the goal of achieving a holistic understanding of cerebellar computation remains a significant challenge.

At the algorithmic level, current evidence suggests at least two different learning rules that the cerebellum can harness to predictively modify its inputs. If the cerebellum can utilize both supervised (Raymond and Medina, 2018) and reinforcement learning strategies (Heffley and Hull, 2019; Heffley et al., 2018; Kostadinov et al., 2019; Larry et al., 2019), it is necessary to understand 1) what are the specific behavioral conditions that determine which learning rule(s) are used, and 2) what are the circuit mechanisms and pathways the enable different learning rules? For example, can any cerebellar behavior motivated by reward consumption utilize CF reinforcement learning signals? And are these reinforcement learning signals computed in the inferior olive, or inherited from upstream brain regions? Such questions highlight the necessity for more detailed anatomical studies of cerebellar input and output pathways, and an understanding of what behaviors specifically engage different pathways.

If the cerebellum does exhibit multiple functionality, the learning rules used may also be area-specific, as it is clear that the cerebellum is functionally compartmentalized (Apps and Garwicz, 2005). Again, human imaging studies provide a crucial basis for generating circuit-based predictions about area-specific processing, and recent work has significantly extended our understanding of both what is processed in different parts of the human cerebellum (King et al., 2019), and where these regions project across the brain (Ramnani et al., 2006). However, caution is warranted in extending these observations to animal models, as the functional homology across species remains incompletely understood. To overcome this issue, further investigation of cross-species circuit homologies is necessary (Sugihara, 2018), as well as work that can clearly define input and output pathways across the cerebella of distinct species.

Finally, at the implementation level, the crystalline cellular architecture of the cerebellum has long suggested uniformity in the basic building blocks for executing cerebellar computation. However, there is also ample evidence that, despite this gross uniformity, there are many regional specializations that shape neuronal excitability, relative density of distinct cell types, molecular marker expression, and other circuit properties (Cerminara et al., 2015). Moreover, recent evidence suggests that unique cell types such as inhibitory interneurons may be selectively engaged to modulate learning (Gaffield et al., 2018; Rowan et al., 2018), and that well-studied plasticity rules thought to underlie learning may in fact be region specific, and tuned to different behaviors (Suvrathan et al., 2016). If plasticity rules differ according to behavior and/or cerebellar region, such findings raise the possibility that implementation may not be a fixed property of cerebellar circuits. At minimum, implementation it is likely to be flexible, as recent evidence has shown that cerebellar-dependent learning can be modulated by behavioral context (Albergaria et al., 2018). Thus, it will be critical to determine what mechanisms can alter the implementation of cerebellar processing. For example, in other brain regions, neuromodulators play a key role in flexibly altering neural circuit processing. And, while the cerebellum receives significant neuromodulatory input, we are only at the early stages of understanding how these systems modify cerebellar processing (Carey et al., 2011; Dieudonné and Dumoulin, 2000; Fleming and Hull, 2019).

To understand whether and how the implementation of cerebellar learning can differ, it will be important to move beyond single cell or single cell-type measurements. In particular, since learning is likely to involve multiple mechanisms across many sites, simultaneous, circuit-wide measurements are necessary, ideally across different behaviors and species to identify common principles. To achieve this, modern population level recordings based on calcium imaging or high-density electrode arrays will be indispensable in order to generate a holistic picture of how cerebellar processing is implemented.

Together, such measurements targeted across multiple levels of analysis will be essential to achieving a comprehensive, circuit-based understanding of how the cerebellum functions as a neuronal prediction machine. And, while the current paradigm shift beyond motor errors has added new complexity to our understanding of cerebellar circuit function, these experiments are sure to ultimately be rewarding.
